# Executive function improvement in response to meta-cognitive training in chronic mTBI / PTSD

**DOI:** 10.3389/fresc.2023.1189292

**Published:** 2023-07-07

**Authors:** J. Kay Waid-Ebbs, Pey-Shan Wen, Tyler Grimes, Somnath Datta, William M. Perlstein, Carol Smith Hammond, Janis J. Daly

**Affiliations:** ^1^ Department of Veterans Affairs (VA), Rehabilitation Research and Development, Brain Rehabilitation Research Center, Gainesville, FL, United States; ^2^ Department of Occupational Therapy, Byrdine F. Lewis College of Nursing and Health Professions, Georgia State University, Atlanta, GA, United States; ^3^ Department of Mathematics and Statistics, University of North Florida, Jacksonville, FL, United States; ^4^ Department of Biostatistics, University of Florida, Gainesville, FL, United States; ^5^ Department of Clinical & Health Psychology, College of Public Health and Health Professions, University of Florida, Gainesville, FL, United States; ^6^ Audiology and Speech Pathology Service, Durham VAMC, Durham, NC, United States; ^7^ General Internal Medicine, Duke University, Durham, NC, United States; ^8^ Department of Physical Therapy, College of Public Health and Health Professions, University of Florida, Gainesville, FL, United States; ^9^ Department of Neurology, School of Medicine, Case Western Reserve University, Cleveland, OH, United States

**Keywords:** traumatic brain injury, post-traumatic stress disorder, executive function, cognition, complex functional tasks, quality of life, life role participation

## Abstract

**Objective:**

We tested Goal Management Training (GMT), which has been recommended as an executive training protocol that may improve the deficits in the complex tasks inherent in life role participation experienced by those with chronic mild traumatic brain injury and post-traumatic stress disease (mTBI/PTSD). We assessed, not only cognitive function, but also life role participation (quality of life).

**Methods:**

We enrolled and treated 14 individuals and administered 10 GMT sessions in-person and provided the use of the Veterans Task Manager (VTM), a Smartphone App, which was designed to serve as a “practice-buddy” device to ensure translation of in-person learning to independent home and community practice of complex tasks. Pre-/post-treatment primary measure was the NIH Examiner, Unstructured Task. Secondary measures were as follows: Tower of London time to complete (cTOL), Community Reintegration of Service Members (CRIS) three subdomains [Extent of Participation; Limitations; Satisfaction of Life Role Participation (Satisfaction)]. We analyzed pre-post-treatment, *t*-test models to explore change, and generated descriptive statistics to inspect given individual patterns of change across measures.

**Results:**

There was statistically significant improvement for the NIH EXAMINER Unstructured Task (*p* < .02; effect size = .67) and cTOL (*p* < .01; effect size = .52. There was a statistically significant improvement for two CRIS subdomains: Extent of Participation (*p* < .01; effect size = .75; Limitations (*p* < .05; effect size = .59). Individuals varied in their treatment response, across measures.

**Conclusions and Clinical Significance:**

In Veterans with mTBI/PTSD in response to GMT and the VTM learning support buddy, there was significant improvement in executive cognition processes, sufficiently robust to produce significant improvement in community life role participation. The individual variations support need for precision neurorehabilitation. The positive results occurred in response to treatment advantages afforded by the content of the combined GMT and the employment of the VTM learning support buddy, with advantages including the following: manualized content of the GMT; incremental complex task difficulty; GMT structure and flexibility to incorporate individualized functional goals; and the VTM capability of ensuring translation of in-person instruction to home and community practice, solidifying newly learned executive cognitive processes. Study results support future study, including a potential randomized controlled trial, the manualized GMT and availability of the VTM to ensure future clinical deployment of treatment, as warranted.

## 1. Introduction

Traumatic Brain Injury (TBI) can initiate a disease process that produces change that continues years after injury, resulting in debilitating chronic TBI symptomatology (1, 2). A mild TBI is classified by the Veterans' Administration as a TBI that results in either an alteration of consciousness up to 24 h or a loss of consciousness of less than 30 min. After an initial mild TBI (mTBI), up to 30% of people may develop persistent symptoms (3, 4) stemming from diffuse axonal injury (5–7) or neuroinflammatory reactions (8, 9). Persistent cognitive deficits after mTBI present barriers to full functional recovery and re-entry into societal roles. Executive dysfunction, poor attention-concentration, and memory difficulties are the most persistent cognitive disabilities faced by TBI survivors (10–12). Attention and executive function are requisite for other cognitive processes that are vital to everyday functioning such as memory, problem solving, language skills, and the cognitive control of behavior. Cognitive deficits due to TBI lead to long-term disability and immense economic burden (13).

Blast-related mTBI in veterans is often more complicated than mTBI in civilians due to the context of the injury, resulting in both psychological and physical trauma (14). Thus, complicating the situation, an estimated 44% of veterans with mTBI also have comorbid PTSD (15). Of veterans with mTBI receiving medical services in the Veterans Health Administration (VHA), the percentage of PTSD is 73% (16).

To date, for veterans with mTBI, there is a lack of evidence to support effectiveness of cognitive treatment. In fact, most published studies were conducted for those with moderate to severe TBI. There are a few cognitive rehabilitation studies that included veterans with mTBI, which reported improvement in symptoms (17–22); but notably in these studies, there was no report of improvement in functional activities or life role participation (i.e., generalization).

To address this limitation, Goal Management Training (GMT) was recommended in the past as a training that may produce real-life functional gains (23). GMT is a promising intervention that leads to an improvement in functional gains, with a small to moderate effects size in civilians with severe TBI (24). Further, in prior work with those having mTBI/PTSD, we pilot-tested GMT for its feasibility, enrolling six participants and using the short form of GMT. In this pilot work, we found that GMT was a feasible and promising intervention for those with mTBI (25) according to a cognitive function measure; we noted importantly, that GMT, indeed, targets executive function for complex real-life tasks (26, 27). In our feasibility testing of GMT in those with mTBI/PTSD, we identified a difficult obstacle in realizing gains in life role participation; this obstacle was a gap between research lab GMT instruction and independent home practice of complex tasks. We observed that veterans with mTBI required support to transfer the metacognitive strategies that were newly learned in the research lab to independent home practice. Therefore, in other preparatory work, we designed, built, and tested the Veteran's Task Manager, a Smartphone application (app) that could be used for that purpose by veterans with mTBI/PTSD. We found that this app was effective, resulting in more successful independent practice and completion of complex tasks at home and other environments (28). In summary, our purpose was based on the following: the dearth of effective treatment strategies for mTBI/PTSD executive dysfunction; the lack of evidence regarding GMT's efficacy in producing gains in life role participation; GMT feasibility in veterans with mTBI/PTSD; and the promise of GMT in civilian severe TBI. Therefore, our purpose was to study response to the long version of GMT conducted in-person in a larger cohort of mTBI/PTSD veterans, provide the Veteran's Task Manager for supported independent home task-practice, and assess change in cognitive task performance and in life role participation in response to treatment.

## 2. Methods

### 2.1. Participants

This study was a single cohort, pre-/post-treatment design. Veterans reporting cognitive deficits to Speech Services were recruited and treated in two sites (North Florida/South Georgia VA Medical Center and Durham, N. Carolina VA Medical Center. Treatment fidelity across the two sites was ensured with methods including the following: manualized GMT was used; intervention was conducted by the site PI from each facility; and weekly meetings were held of the two PI's, covering review of GMT weekly session content. Research was conducted with the oversight of the Internal Review Boards from the University of Florida (#201601606) and Durham VA Medical Center (#01887).

Subject inclusion criteria were as follows: diagnosis of a blast-related mTBI documented in the medical record by a physician or neuropsychologist; frontal lobe-based cognitive impairment as determined by more than one standard deviation below the mean for the matching age group from the technical manual (29) on trial 3 or 4 of the Delis-Kaplan Executive Function System, Color-Word Interference Test (30); 18 to 55 years old; at least 6-months post injury; no history of pre-morbid learning disability; no psychiatric diagnosis sufficiently severe to have resulted in inpatient hospitalization or a neurological disease unrelated to TBI; score >90 on National Adult Reading Test, Estimated IQ (31); passing score of the validity testing on the Test of Memory Malingering (32); no alcohol or substance abuse within the past year; not involved in litigation; fluent in English.

### 2.2. Intervention

#### 2.2.1. GMT sessions

We conducted ten in-person sessions of training (33) to improve task planning and problem solving, which included the addition of one family education session. Details of each session and homework are included in Table 1. Family/caregiver involvement was not targeted beyond session 1. We administered interactive Power Point modules. Each session was based on a five-stage planning and problem-solving strategy that the learner incorporated into a variety of clinical simulation tasks under the guidance of the therapist. Training was also customized. That is, participants identified three complex functional tasks with which they were having the greatest difficulty; examples included meal planning and shopping; detailing a car; building a birdhouse; or paying monthly bills. The therapist then provided guided support in the practice of task components and whole task performance.

**Table 1:** Content of intervention^a^

Sessions	Content of Goal Management Training (GMT) with modifications	Clinic Simulation Tasks	Homework
2-hour/10-weeks (20 h, total)			20-hours total
Family Education	Mechanisms of blast injury, factors that affect cognition, typical symptoms, recovery and what to expect in treatment	Share symptoms	Monitor sleep, exercise, caffeine intake and write goals (long term, short-term and status). Identify 3 functional tasks to complete using Veterans’ Task Manager (VTM) Smartphone App.
Absent- and Present- Minded	Defining clear objective goals, absent-minded consequences of action slips	Clapping Task	Monitor absent-minded slips
Absent- Minded Slip-ups	Relationship of absent-mindedness to other abilities, conditions that make slips more likely	Clapping Task	Monitor absent-minded slips and consequences; Practice Body Scan
		Body Scan	
		^b^Mindfulness Video #1	
“Automatic Pilot”	Automatic Pilot can lead to errors	Card Dealing Task	Monitor Present Mindedness; Practice Breathing Exercise
		Complex task I	
		^b^Mindfulness Video #2	
Stop Automatic Pilot	Stop the automatic pilot and making STOPPING a habit.	Using “STOP” during Clapping and Card Tasks	Monitor stopping during functional tasks; Practice breath and focus.
		Breath and Focus	Set-up and complete functional task I, using the Veterans Task Manager App.
		Complex task II	
		^b^Mindfulness Video #3	
Mental Blackboard	Using “STOP” to check your mental blackboard/working memory. What am I supposed to be doing? STOP, BREATH FOCUS, CHECK	Stop-focus-check	Monitor Stop-Focus-Check-what am I supposed to be doing?
		Dual task with card sorting and recognizing identical words	Longer Breathing Exercise
		Complex task III.	Catalog Task I: buy gifts with constraints while staying within a budget.
		^b^Mindfulness Video #4	
State Your Goal	Refresh goal by saying stop, checking mental blackboard and stating goal out loud. STOP, BREATH FOCUS, RE-STATE GOAL.	Review Catalog Task I	Monitor 30-minute practice of Stop-State cycle; longer breathing exercise and slips and successes; Catalog Task II.
		^b^Mindfulness Video #5	Set-up and complete functional task II, using the Veterans Task Manager App.
Making Decisions	Goal conflict and decision making. When stressed STOP, BREATHE to reduce stress and REFOCUS, STATE goal. Incorporate a “to do list” to remember plan. Indecision, “just do it” and love your decision.	Bookkeeping Task I	Monitor STOP, STATE and Breathing exercises. Catalog Task III.
Splitting Tasks into Subtasks	Break an overwhelming task into sub-goals and steps. STOP-STATE-SPLIT cycle	Bookkeeping Task II.	Monitor STOP, STATE, SPLIT and Breathing Exercises.
			Set-up and complete functional task III, using the Veterans Task Manager App.
Checking (Stop!)	Use to do list, notes and alarms to reduce forgetting and make STOP at regular intervals a habit. STOP, FOCUS, CHECK and STOP STATE SPLIT are reviewed.	Clapping Task	
		Bookkeeping Task III	

^a^ Content is derived from published GMT modules (33).

Underlined content: moved to different time points vs. that in published GMT modules.

VTM, Veterans Task Manager is available in Google Play and the Apple Store.

Bold content: additional activities not contained in the published GMT modules.

^b^ Mindfulness videos: available on YouTube and were developed by Wolf, C. & Serpa, JG (34).

#### 2.2.2. Veterans task manager

We provided participants with the Veteran's Task Manager, a Smartphone application (app; VTM), which we developed and implemented in order to specifically practice the problem-solving steps taught in GMT. The Veteran's Task Manager provided the following learning-assist features: simplify tasks to small components; estimate time to complete; track and identify each step as completed (check list); and a visual/vibrating alert to stay “On Target”. The Veteran's Task Manager is available in both Apple and Google Smartphone app stores.

### 2.3. Primary measure

#### 2.3.1. Unstructured task (from the NIH executive abilities: measures and instruments for neurobehavioral evaluation and research (EXAMINER)

The Unstructured Task was selected as the primary measure because, not only is it a standardized test, but also it requires attention and executive function processes required for the complex tasks of life role participation and processes which are targeted in GMT. The test required users to complete paper and pencil problems of varying difficulty levels within 6 min (35). A higher score indicates better performance, with a maximum score of 1,469 points. The Unstructured Task has a test re-test reliability of .71 and separates neurological patients from controls (F = 11.2, *p* < .005) (36).

### 2.4. Secondary measures

#### 2.4.1. Time to complete, from the computerized Tower of London (cTOL)

The cTOL is a measure of the speed of problem-solving required to solve a multiple-step, visual-spatial problem, which demands problem-solving while keeping the final goal in mind (37), which are features inherent in complex task completion. In prior work, we found that cTOL test-retest reliability within one week was .85 in those with mTBI (38).

#### 2.4.2. Community reintegration of service members (CRIS)

The CRIS is a measure of life role participation activities, developed specifically for soldiers with disabilities (39–41). Each of the three CRIS subdomains have good internal reliability, as follows: Extent of Participation = 0.91, Perceived Limitations = 0.93, and Satisfaction with Participation = 0.97 (39). Minimum detectable change (MDC) indices were 5.9, 6.2, and 3.6, respectively (42).

#### 2.4.3. Sample characteristics

We characterized the sample according to depression, which was measured using the Beck Depression Inventory (BDI-II), a 21-item questionnaire (43, 44), with the following categories: total scores of 0–13 minimal depression; 14–19 mild; 20–28 moderate; 29–63 severe. PTSD symptom severity was assessed based on the PTSD symptom checklist-military (PCL-M) (45, 46).

### 2.5. Data analysis

For the NIH EXAMINER Unstructured Task, cTOL and CRIS variables, we conducted a pre-/post-treatment paired *t*-test to obtain *p*-values for assessing statistical significance, calculated effect sizes, and computed 95% confidence interval using the bias-corrected and accelerated (BCa) bootstrap method (47). Effect size was calculated by dividing the change score by the pooled estimate for standard deviation of the outcome (48). The statistical software R was used to conduct analyses and create figures (49). If there was a missing data point, the participant data was removed from analysis. Because the sample size was small, we conducted exploratory analyses. We used IBM SPS Statistics v28.0.0.0 to analyse Pearson correlation models investigating any potential relationship between gain in the Unstructured Task outcome measure and characteristics of depression, IQ, and PTSD score. We conducted the same correlation analysis for the cTOL outcome measure and subject characteristics. Descriptive analysis was conducted including the range of scores and the percent of participants showing change; and scatterplots were generated for visual inspection.

## 3. Results

### 3.1. Participants

Forty-eight participants were screened of which 19 did not meet inclusion criteria and 3 declined to participate after testing. Subsequently, there were 12 dropouts [starting a new job (*n* = 2)]; demands at school increased (*n* = 2); moving (*n* = 1); new baby (*n* = 1); coming to clinic increased anxiety (*n* = 3); and no reason given (*n* = 3). Thus, 14 participants completed GMT (8 at the NF/SG site and 6 at the Durham site). Subject characteristics are given in Table 2. All participants, except one (Subject 13), had a PTSD diagnosis. All participants exhibited some signs of depression, with scores ranging from 12 (Subject 13) to 54 (Subject 4).

**Table 2:** Participant characteristics.

Characteristics	Mean ± standard deviation
Age	38.4 ± 7.2
Years Education	14.2 ± 1.5
Years from Last mTBI	9.4 ± 4.6
Number of Blast Exposures	4.9 ± 7.9
Race	9 (64%) White
	4 (29%) Black
	1 (7%) Native Hawaiian
Vocational Status	7 (50%) Unemployed
	2 (14%) Full-Time Employment
	5 (36%) Full-Time Student Stipend
Estimated Intelligence Quotient	111.1 ± 7.02
	(Range 97–122)
Beck's Depression Inventory-II	31 ± 12.2
	(Range 12–54)
PTSD Checklist-Military Version	60.9 ± 16.4
	(Range 26–85)
	Only 1 participant did not exhibit PTSD (that is, <30)

### 3.2. Improvement in executive function according to standardized tasks

#### 3.2.1. Primary measure

There was statistically significant improvement for the NIH EXAMINER Unstructured Task (*p* = 0.02; effect size = .67; Table 3). One subject was lost to follow-up at post-testing; therefore, that participant data was removed from analysis, with remaining sample size, *n* = 13.

**Table 3.** Significant Improvement at Post-Treatment According to Primary and Secondary Measures of Executive Cognitive Function and Life Role Participation

Measure	Pre-treatment Mean (SD)	Post-treatment Mean (SD)	Mean Difference (SD)	95% CI	*p*-value	Effect size
**EXAMINER Unstructured Task** (*n* = 13)	355 (94.3)	417 (96.1)	62.8 (84.3)	(28, 120)	**0.02** *	0.67
**cTOL Time to Completion** (*n* = 13)	24.7 (5.6)	21.7 (4.3)	−2.9 (3.6)	(−5.69, −1.60)	**0.01** *	0.52
**CRIS** Extent of Participation In Life Roles (*n* = 9)	36.0 (7.5)	41.6 (4.3)	5.6 (4.6)	(2.75, 8.39)	**0.01** *	0.75
**CRIS** Perceived Limitations In Life Role Functions (*n* = 9)	38.2 (7.5)	42.7 (6.3)	4.4 (5.7)	(0.49, 7.60)	**0.05** *	0.59
**CRIS** Satisfaction with Participation in Life Role (*n* = 9)	36.1 (12.0)	40.0 (7.1)	3.9** (8.1)	(−1.02, 9.15)	0.18	0.33

Key:

SD=standard deviation

* bold = statistically significant p value

** Minimum Clinically Important Difference (MCID, CRIS measure; 40)

#### 3.2.2. Secondary measure

cTOL. There was statistically significant improvement for the Tower of London, Time to Completion (*p* = 0.012; effect size = .52; Table 3). One subject was lost to follow up at post-testing; therefore, that participant data was removed from analysis, with remaining sample size, *n* = 13.

### 3.3. Improvement in life role participation

CRIS. There was statistically significant improvement in Life Role Participation, as follows: Extent of Participation in life roles (*p* < .01; effect size = .75; Table 3) and Perceived Limitations in life role participation (*p* < .05; effect size = .59; Table 3). The Satisfaction in life roles participation subdomain did not reach significance (*p* = .18; Table 3). We encountered difficulties with the available software for the CRIS, and subsequently five data points for the CRIS were lost, for which those participants were removed from analysis, with the remaining analysed sample size of 9 participants.

### 3.4. Exploratory correlation analyses and descriptive analyses

There was no significant relationship between the cTOL and either depression, PTSD symptom severity, or IQ (*r* = .04, *p* = .45; *r* = .116, *p* = .35; *r* = .036. *p* = .45, respectively). There was no significant relationship between the Unstructured Task and either depression or IQ (*r* = .12, *p* = .31; *r* = .15, *p* = .35, respectively). There was a relatively higher, but non-significant correlation between the Unstructured Task and PTSD severity (*r* = .439, *p* = .07). These results should be interpreted with some caution, due to the small sample size of the study.

**Figure 1:**
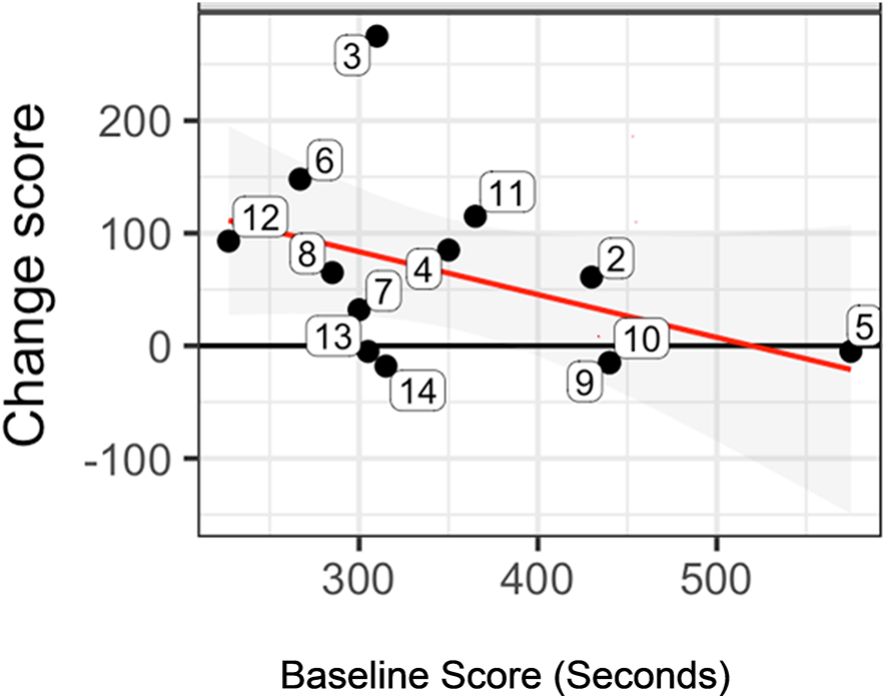
Statistically significant pre-/post-treatment change score for the NIH examiner unstructured task. Figure 1 shows the change score for each subject (numbered bubbles), according to The Examiner unstructured task. In general, those with a lower baseline score showed greater improvement in change score in comparison with those with higher baseline scores (negative slope of the regression line (red line). The horizontal axis is the pre-treatment score and the vertical axis is the change score. Key: Black horizontal line (y = 0) represents no change from postminus pre-treatment scores. Red regression line summarizes the conditional change at post-treatment given the pre-treatment measure. Gray shaded area shows the 95% confidence interval associated with the red regression line.

Data inspection for individuals showed that 93% of participants (13/14) had improvement in one or more outcome measures, in terms of score change. The NIH Examiner Unstructured Task showed that 67% of the participants showed a gain in score (Figure 1); in general, those with a lower baseline score showed greater improvement in change score in comparison with those with higher baseline scores (negative slope of the regression line (red line; Figure 1).

**Figure 2:**
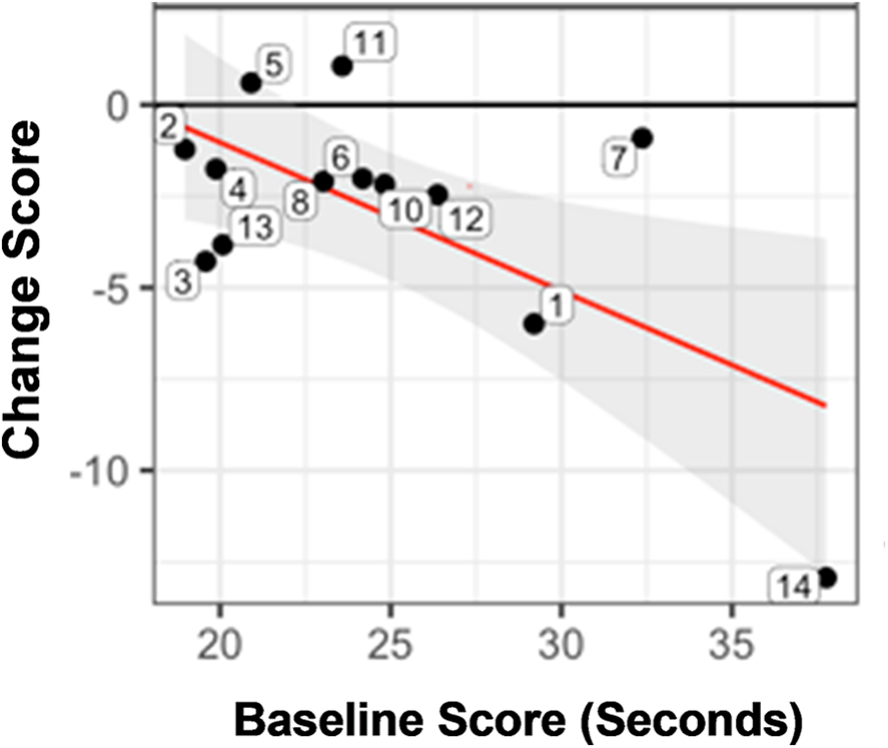
Statistically significant pre-/post-treatment change score for the time to completion for the tower of London (cTOL). Figure 2 shows the change score for each subject (numbered bubbles), according to the time to complete the tower of London (cTOL). The improvement in score (i.e., higher negative number for the change score) was, in general, greater for those with a worse baseline performance (longer time to complete at pre-treatment). The horizontal axis is the pre-treatment score and the vertical axis is the change score. Key: Black horizontal line (y = 0) represents no change from post minus pre-treatment scores. Red regression line summarizes the conditional change at post-treatment given the pre-treatment measure. Gray shaded area shows the 95% confidence interval associated with the red regression line.

The cTOL showed that 77% had a gain in score (Figure 2). The improvement in score (i.e., higher negative number for the change score) was, in general, greater for those with a worse baseline performance (longer time to complete at pre-treatment). An example of this is subject 14 (lower, right corner of Figure 2), who had the greatest change score (improvement) at post-treatment, but began at pre-treatment with the longest time (worst performance) to completion at pre-treatment.

**Figure 3:**
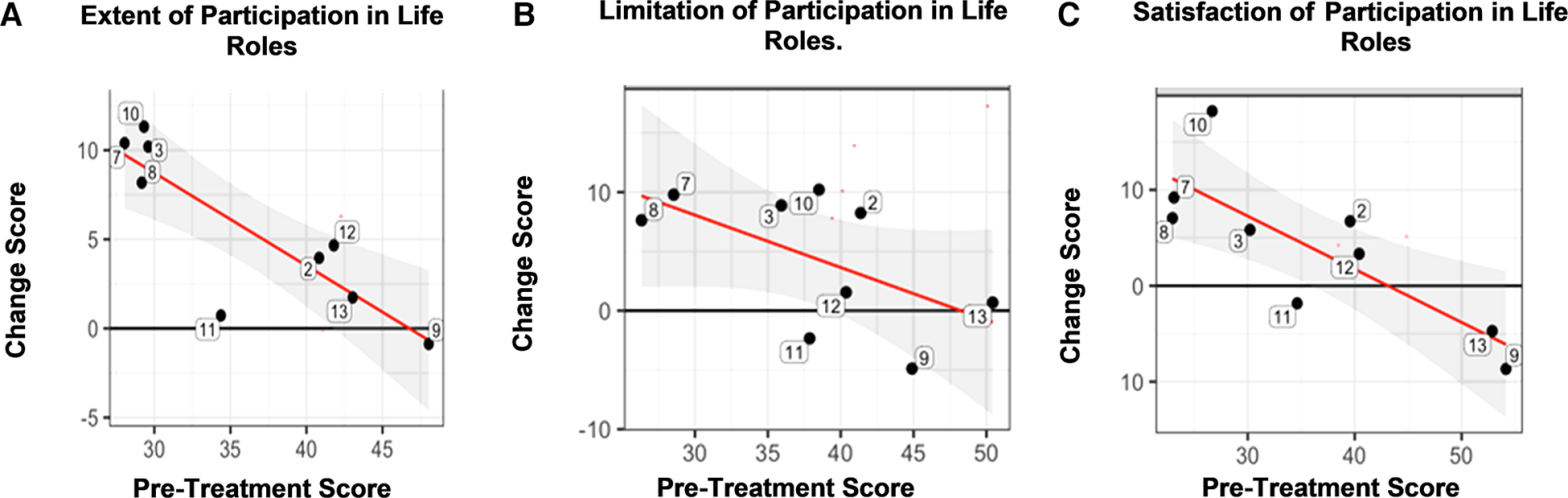
Community Reintegration of Service Members (CRIS). Panel A. There was a statistically significant improvement according to the CRIS subdomain of Extent of Participation in Life Roles, which showed that, in general, those with the lowest baseline score (horizontal axis), showed greater improvement (vertical axis) in comparison to those with higher baseline scores. The horizontal axis is the pre-treatment score and the vertical axis is the change score. Panel B. There was a statistically significant improvement according to the CRIS subdomain of Limitation in Life Role Participation, which showed that, in general, those with the lowest baseline score (horizontal axis), showed greater improvement (vertical axis) in comparison to those with higher baseline scores. The horizontal axis is the pre-treatment score and the vertical axis is the change score. Panel C. The CRIS subdomain of Satisfaction in Life Role Participation showed that, in general, those with the lowest baseline score (horizontal axis), showed greater improvement (vertical axis) in comparison to those with higher baseline scores. The horizontal axis is the pre-treatment score and the vertical axis is the change score. Key: Black horizontal line (*y* = 0) represents no change from post- minus pre-treatment scores. Red regression line summarizes the conditional change at post-treatment given the pre-treatment measure. Gray shaded area shows the 95% confidence interval associated with the red regression line.

For the CRIS, 89% of participants improved according to the Extent of Participation in life roles (Figure 3, Panel A). In general, those with the lowest baseline score (horizontal axis), showed greater improvement (vertical axis; Figure 3, Panel A). For the CRIS Perceived Limitations, 78% of participants improved (Figure 3, Panel B). On the CRIS subdomain of Satisfaction, 67% improved (Figure 3, Panel C). In terms of the MDC for the CRIS Satisfaction in Life Roles Participation, the group mean gain score exceeded the MDC threshold (Table 3), indicating a mathematically detectable, and therefore, meaningful difference. This occurred even though three veterans, who had high baseline scores, did not show improvement. For the CRIS Extent of Participation in Life Roles, the group mean gain score closely approached the minimal detectable change (MDC), just shy of the threshold by 0.03 points (Table 3).

In terms of individuals, subject 13 showed the least depression, the least severe PTSD symptomatology, and was in the top quartile of treatment response according to the cTOL (for example, Figure 2); and subject 4 showed the worst depression, the greatest PTSD symptomatology, and was close to the bottom quartile of treatment response according to the cTOL. There was only one subject in the group who did not show some improvement across measures (Subject 9; for example, almost no change, Figure 1); he had the second least depression score (14 points), the second least PTSD symptoms (44 points), and the highest IQ (122).

## 4. Discussion

This study contributes to the literature in a number of ways. First, we focused on veterans with mTBI/PTSD and their need for executive function training. Second, we produced statistically significant improvement in life role participation in response to the executive training methods. Third, we designed the intervention to include advantages and support required to translate learning from the research lab to the home and living environments. That is, we administered the long form of GMT for more intensive instruction and learning; and we provided the Veteran's Task Manager to participants for support and transfer of newly learned strategies to independent practice at home and other environments. A fourth contribution is that we showed that the Examiner Unstructured Task was sensitive in capturing pre-/post-treatment improvement in those with mTBI/PTSD. Fifth, descriptive analyses provided insight into individual response to treatment, supporting the need for precision neurorehabilitation.

### 4.1. Focus on veterans with mTBI/PTSD

The dearth of executive function training studies on behalf of mTBI/PTSD is likely due to the inherent difficult challenges. Those challenges include persistent executive dysfunction, poor attention-concentration, and memory difficulties preventing re-entry into societal roles (50, 51). These challenges are compounded in the 13%–33% of Veterans who have both mTBI and PTSD (52–54). The neural disruptions shared by PTSD and TBI include asymmetrical white matter tract abnormalities and gray matter changes in the basolateral amygdala, hippocampus, and prefrontal cortex (55, 56). For both mTBI and PTSD, disruptions occur in the fronto-congulo-parietal cognitive control network circuit, various executive function domains such as the anterior cingulate cortex involving cognitive control, the dorsolateral prefrontal cortex mediating working memory, the inferior frontal gyrus and (pre-) supplementary motor area regulating response inhibition, and the parietal lobes involving attention and its control (55, 57).

There are few studies reporting consistently convincing evidence on the effectiveness of executive function training (58), and studies on the mTBI and PTSD comorbid condition are scarce. A few recent reports documented modest improvement from cognitive training in some areas of cognitive functioning in military members and Veterans with mTBI (17–22). Although promising, there were limitations as follows: intervention that lacked training of cognitive processes essential to executive function (17), lacked sufficient training time (18, 21, 22), or training time for cognitive components was not reported (18, 20). The current study addresses some of these limitations by having enrolled and treated those with mTBI/PTSD. Therefore, this study provides preliminary results on which to further develop evidence-based practice for clinical care, given that “…symptoms from each [condition (mTBI or PTSD)] may be often indistinguishable suggesting that assessment and treatment of mTBI and PTSD benefits from better clinical integration” (59).

### 4.2. Significant improvement in life role participation for those with mTBI/PTSD in response to GMT

In a prior meta-analysis of GMT studies (60), no studies reported measures of community participation. Life role participation is the ultimate goal of rehabilitation (61). It is critical to develop interventions that can effect change and improvement in life role participation for veterans with mTBI/PTSD. In the current study, GMT resulted in sufficiently robust improvement in executive function so as to produce significant improvement in life role and community participation.

### 4.3. Intervention designed for advantageous incremental, customized learning and support required to translate new learning to the home and community activities

A prior meta-analysis of other GMT studies (60) combined multiple diagnoses of brain injury (ABI, MS, ADHD, CVD, Spina Bifida, SUD) but included only two studies (*n* = 2/33 studies) of those with mild TBI). In their analysis, they accepted studies with multiple outcome measures of executive function including the following: Multiple Errands Test, Hotel Task, and BADS Zoo task. From this information, they reported that their meta-analysis showed a small effect size (Hedges' g = 0.297) immediately following training. Their meta-analysis is an important study as a beginning investigation of GMT across patient diagnostic categories. In fairness, our relatively stronger results cannot be directly compared to the meta-analysis described above (59) because our sample was composed of one diagnostic category compared to their multiple diagnoses; further, our measures were selected according to their ability to assess the cognitive processing and task performance that was practiced during GMT. These study design differences could explain, in part, the higher effect sizes in the current study [Unstructured Task and Extent of Participation in Life Roles (Cohen's d = 0.67, and.75, respectively)].

Additionally, the promising results of the current study could be the function of a number of treatment protocol features. First, the GMT protocol itself encourages the learner to identify three complex tasks that they care about performing and with which they are having difficulty. This feature is an example of precision (customized) neurorehabilitation. A second advantageous feature of GMT is the instruction regarding division of a complex activity into main goal, subgoals, and action steps; this process is guided for success by the therapist, if needed. A third treatment advantage was the use of the VTM app, used for home practice as a memory aid, for keeping in mind the main goal, subgoals, and action steps, during a given activity. Fourth, the VTM provided an alert signal to assist with focus of attention on a given action step. Fifth, the VTM provided a feature that may have improved ability to plan the time for a given task; that is, the VTM timer was used for assessing task completion compared to the prior-predicted time given by the user.

Given these strongly supportive features of the GMT itself and the use of the VTM, it is reasonable to consider that they had a strong impact on the gains in response to treatment.

### 4.4. Significant improvement according to both examiner unstructured task and time to completion of the Tower of London (cTOL)

Our results showed that the NIH Examiner Unstructured Task was sensitive to change in those with mTBI/PTSD in response to GMT (*p* = .02). The EXAMINER Unstructured Task presents a problem to the user, which is a simulation of a functional task requiring cognitive processes and strategies similar to those cognitive processes and strategies instructed and practiced during GMT task training. These include mindfulness, stating the main goal, generating a plan and evaluating results. In severe TBI, GMT has been offered, with reports of significant improvement in measures, such as the Hotel Task, the Modified Multiple Errands Task and the Zoo Map Test (62, 63). These studies were for severe TBI and the measures are specific to a particular environment(s). In the current study, we contributed to the literature by enrolling mTBI/PTSD, and we used the NIH Examiner Unstructured Task, which assesses cognitive processing for complex tasks using a standardized test, with relevance to any environment.

In the current study, GMT significantly improved cTOL (time to completion), indicating improved efficiency. These prior studies reported varying results, according to TOL variables. One study reported no improvement (64). The other two studies enrolled severe TBI and reported significant improvement on two TOL variables, the “TOL achievement score” and the ‘TOL rule violation score (65, 66). The achievement score is the sum of nine puzzles that are correctly built within the time frame with the least number of moves possible; the rule violation score is the number of times the participant picks up more than one disk at a time or places a larger disc onto a smaller disc. Both studies had significant improvement on the achievement score and rule violations (65, 66). We found significant gain in cTOL in a prior study (25) and in the current study (*p* < .01).

### 4.5. Individual response to treatment

Our descriptive data showed varied results that potentially support the individual nature of treatment response in those with mTBI and PTSD and thus, the need for precision neurorehabilitation. First, for example, for the group, the negative slope of the regression line for the Unstructured Task (Figure 1) shows the general trend that those with lower baseline scores showed greater change in response to treatment vs. those with higher baseline scores. Second, and in contrast, there was little to no relationship between executive function gains and depression level, PTSD severity, and IQ, according to group correlation analyses. Third, however, two subjects at the extremes of either high symptom severity or low symptom severity showed least or most improvement, respectively, as might be expected. There may be a complex interaction of characteristics and treatment response, given the expected qualitative individual results for these two subjects, along with possible baseline influence (for example, Figure 1), as well as the absence of group correlation with symptomatology and IQ. These results do appear to call for a larger future study to explore individual characteristics and their contribution to treatment response. We designed the intervention according to precision medicine intervention criteria specifically because of the understanding of the individually unique constellation of symptoms, the requirements of GMT itself, the current dearth of literature regarding effective executive function intervention, and improvement for those with mTBI and PTSD. Therefore, we were able to administer treatment that was customizable; taking into account the constellation of symptoms for a given individual. Our results showed that the GMT protocol along with the VTM practice “buddy” could address the unique complexities of mTBI/PTSD, while treating the individual, even in the presence of the obstacles inherent in the complexities of pathologies and cognitive impairments in mTBI/PTSD. Because the GMT is manualized and the VTM is available as a Smartphone app, this combined intervention could, as warranted, be easily deployed to clinical practice.

### 4.6. Study limitation

In this single-cohort study, we would like to note a number of limitations. First, the sample size was small, indicating the standard limitations of generalization of the results. For example, a larger sample size should be studied regarding the influence of individual characteristics on treatment response, including the correlation analyses provided here. Second, given the heterogeneous nature of TBI, there are limitations to generalization of these findings. Third, in this study, we acquired and report two secondary measures, without multiple *p*-value corrections; therefore, these results should be considered in that light. Fourth, we did not acquire follow-up data at a time point following the end of treatment. At the same time, given the need and the promising results, a larger randomized, controlled trial can be justified for GMT with Veterans Task Manager for those with mTBI and PTSD.

## 5. Conclusions

In Veterans with mTBI/PTSD in response to GMT and the VTM learning support buddy, there was significant improvement in executive cognition processes sufficiently robust to produce significant improvement in community life role participation. There was individual variation in treatment response, indicating the need for precision neurorehabilitation in those with mTBI/PTSD. The positive results occurred likely in response to treatment advantages afforded by the content of the combined GMT and the employment of the VTM learning support buddy, with advantages including the following: manualized content of the GMT; incremental complex task difficulty; GMT structure and flexibility to incorporate individualized functional goals; and the VTM capability of ensuring translation of in-person instruction to home and community practice, solidifying newly learned executive cognitive processes. Study results support future study, including a potential randomized controlled trial. The manualized GMT and availability of the VTM ensure future clinical deployment of treatment, as warranted.

## Data Availability

The raw data supporting the conclusions of this article will be made available by the authors, without undue reservation.

## References

[1] Masel BE DeWitt DS. Traumatic brain injury: a disease process, not an event. J Neurotrauma. (2010) 27(8):1529–40. 10.1089/neu.2010.1358 20504161

[2] Whitnall L McMillan TM Murray GD Teasdale GM. Disability in young people and adults after head injury: 5–7 year follow up of a prospective cohort study. J Neurol Neurosurg Psychiatry. (2006) 77(5):640–5. 10.1136/jnnp.2005.078246 16614025 PMC2117429

[3] Rickels E von Wild K Wenzlaff P. Head injury in Germany: a population-based prospective study on epidemiology, causes, treatment and outcome of all degrees of head-injury severity in two distinct areas. Brain Inj. (2010) 24(12):1491–504. 10.3109/02699052.2010.498006 20645706

[4] Zumstein MA Moser M Mottini M Ott SR Sadowski-Cron C Radanov BP Long-term outcome in patients with mild traumatic brain injury: a prospective observational study. J Trauma. (2011) 71(1):120–7. 10.1097/TA.0b013e3181f2d670 21045743

[5] Mac Donald CL Johnson AM Cooper D Nelson EC Werner NJ Shimony JS Detection of blast-related traumatic brain injury in U.S. military personnel. N Engl J Med. (2011) 364(22):2091–100. 10.1056/NEJMoa1008069 21631321 PMC3146351

[6] Hellyer PJ Leech R Ham TE Bonnelle V Sharp DJ. Individual prediction of white matter injury following traumatic brain injury. Ann Neurol. (2013) 73(4):489–99. 10.1002/ana.23824 23426980

[7] Kinnunen KM Greenwood R Powell JH Leech R Hawkins PC Bonnelle V White matter damage and cognitive impairment after traumatic brain injury. Brain. (2011) 134(Pt 2):449–63. 10.1093/brain/awq347 21193486 PMC3030764

[8] Mayer AR Mannell MV Ling J Gasparovic C Yeo RA. Functional connectivity in mild traumatic brain injury. Hum Brain Mapp. (2011) 32(11):1825–35. 10.1002/hbm.21151 21259381 PMC3204375

[9] Bazarian JJ Zhong J Blyth B Zhu T Kavcic V Peterson D. Diffusion tensor imaging detects clinically important axonal damage after mild traumatic brain injury: a pilot study. J Neurotrauma. (2007) 24(9):1447–59. 10.1089/neu.2007.0241 17892407

[10] Belanger HG Vanderploeg RD Curtiss G Warden DL. Recent neuroimaging techniques in mild traumatic brain injury. J Neuropsychiatry Clin Neurosci. (2007) 19(1):5–20. 10.1176/jnp.2007.19.1.5 17308222

[11] Bogdanova Y Verfaellie M. Cognitive sequelae of blast-induced traumatic brain injury: recovery and rehabilitation. Neuropsychol Rev. (2012) 22(1):4–20. 10.1007/s11065-012-9192-3 22350691 PMC4372457

[12] Vasterling JJ Verfaellie M Sullivan KD. Mild traumatic brain injury and posttraumatic stress disorder in returning veterans: perspectives from cognitive neuroscience. Clin Psychol Rev. (2009) 29(8):674–84. 10.1016/j.cpr.2009.08.004 19744760

[13] Humphreys I Wood RL Phillips CJ Macey S. The costs of traumatic brain injury: a literature review. ClinicoEconomics and Outcomes Research: CEOR. (2013) 5:281–7. 10.2147/CEOR.S44625 23836998 PMC3699059

[14] McDonald S Gowland A Randall R Fisher A Osborne-Crowley K Honan C. Cognitive factors underpinning poor expressive communication skills after traumatic brain injury: theory of mind or executive function? Neuropsychology. (2014) 28(5):801–11. 10.1037/neu0000089 24819067

[15] Hoge CW McGurk D Thomas JL Cox AL Engel CC Castro CA. Mild traumatic brain injury in U.S. Soldiers returning from Iraq. N Engl J Med. (2008) 358(5):453–63. 10.1056/NEJMoa072972 18234750

[16] Taylor BC Hagel EM Carlson KF Cifu DX Cutting A Bidelspach DE Prevalence and costs of co-occurring traumatic brain injury with and without psychiatric disturbance and pain among Afghanistan and Iraq war veteran V.A. Users. Med Care. (2012) 50(4):342–6. 10.1097/MLR.0b013e318245a558 22228249

[17] Nelson LA Macdonald M Stall C Pazdan R. Effects of interactive metronome therapy on cognitive functioning after blast-related brain injury: a randomized controlled pilot trial. Neuropsychology. (2013) 27(6):666–79. 10.1037/a0034117 24059443

[18] Twamley EW Jak AJ Delis DC Bondi MW Lohr JB. Cognitive symptom management and rehabilitation therapy (CogSMART) for veterans with traumatic brain injury: pilot randomized controlled trial. J Rehabil Res Dev. (2014) 51(1):59–70. 10.1682/JRRD.2013.01.0020 24805894

[19] Cooper DB Bowles AO Kennedy JE Curtiss G French LM Tate DF Cognitive rehabilitation for military service members with mild traumatic brain injury: a randomized clinical trial. J Head Trauma Rehabil. (2017) 32(3):E1–E15. 10.1097/HTR.0000000000000254 27603763

[20] Huckans M Pavawalla S Demadura T Kolessar M Seelye A Roost N A pilot study examining effects of group-based cognitive strategy training treatment on self-reported cognitive problems, psychiatric symptoms, functioning, and compensatory strategy use in OIF/OEF combat veterans with persistent mild cognitive disorder and history of traumatic brain injury. J Rehabil Res Dev. (2010) 47(1):43–60. 10.1682/JRRD.2009.02.0019 20437326 PMC4755481

[21] Storzbach D Twamley EW Roost MS Golshan S Williams RM O'Neil M Compensatory cognitive training for operation enduring freedom/operation Iraqi freedom/operation new Dawn veterans with mild traumatic brain injury. J Head Trauma Rehabil. (2017) 32(1):16–24. 10.1097/HTR.0000000000000228 27022961

[22] Vas A Chapman S Aslan S Spence J Keebler M Rodriguez-Larrain G Reasoning training in veteran and civilian traumatic brain injury with persistent mild impairment. Neuropsychol Rehabil. (2016) 26(4):502–31. 10.1080/09602011.2015.1044013 26018041

[23] Cicerone KD. Participation after multidisciplinary rehabilitation for moderate to severe traumatic brain injury in adults. Arch Phys Med Rehabil. (2013) 94(7):1421–3. 10.1016/j.apmr.2013.04.003 23800407

[24] Krasny-Pacini A Chevignard M Evans J. Goal management training for rehabilitation of executive functions: a systematic review of effectiveness in patients with acquired brain injury. Disabil Rehabil. (2014) 36(2):105–16. 10.3109/09638288.2013.777807 23597002

[25] Waid-Ebbs JK Bcba D Daly J Wu SS Berg WK Bauer RM Response to goal management training in veterans with blast-related mild traumatic brain injury. J Rehabil Res Dev. (2014) 51(10):1555–66. 10.1682/JRRD.2013.12.0266 25860148

[26] Levine B Robertson IH Clare L Carter G Hong J Wilson BA Rehabilitation of executive functioning: an experimental-clinical validation of goal management training. J Int Neuropsychol Soc. (2000) 6(3):299–312. 10.1017/S1355617700633052 10824502

[27] Chen AJ Novakovic-Agopian T Nycum TJ Song S Turner GR Hills NK Training of goal-directed attention regulation enhances control over neural processing for individuals with brain injury. Brain. (2011) 134(Pt 5):1541–54. 10.1093/brain/awr067 21515904 PMC6410956

[28] Waid-Ebbs JK. In: Waid-Ebbs JK, editors. Archival veteran's task manager completion data. Gainesville, FL (2023).

[29] Delis DC Kaplan E Kramer JH. The DelisKaplan executive function system: Technical manual. San Antonio: The Psychological Corporation (2001b).

[30] Delis DC Kramer JH Kaplan E Holdnack J. Reliability and validity of the delis-kaplan executive function system: an update. J Int Neuropsychol Soc. (2004) 10(2):301–3. 10.1017/S1355617704102191 15012851

[31] Nelson HE Willison J. National adult Reading test (NART). Ontario, Canada: Nfer-Nelson Windsor (1991).

[32] Tombaugh TN. Test of memory malingering: tOMM: multy-health systems. North Tonowanda, NY: Multi-health Systems (1996).

[33] Levine B Manly T Robertson IH. Goal management training, trainer’s manual. Toronto, ON, Canada: Baycrest Centre for Geriatric Care (2012).

[34] Wolfe CS. Mindfulness Videos (Available at: https://youtu.be/JbGe9BpniJo?t=18) (2023).

[35] Kramer JH Mungas D Possin KL Rankin KP Boxer AL Rosen HJ NIH EXAMINER: conceptualization and development of an executive function battery. J Int Neuropsychol Soc. (2014) 20(1):11. 10.1017/S1355617713001094 24103232 PMC4474183

[36] Kramer JH. EXAMINER User Manual 3.6 (2014).

[37] Berg WK Byrd DL McNamara JP Case K. Deconstructing the tower: parameters and predictors of problem difficulty on the tower of London task. Brain Cogn. (2010) 72(3):472–82. 10.1016/j.bandc.2010.01.002 20167413

[38] Waid-Ebbs JK. Test Retest Reliability of cTOL in five Veterans with mTBI (2023).

[39] Resnik L Plow M Jette A. Development of CRIS: measure of community reintegration of injured service members. J Rehabil Res Dev. (2009) 46(4):469–80. 10.1682/JRRD.2008.07.0082 19882482 PMC3517188

[40] Resnik L Gray M Borgia M. Measurement of community reintegration in sample of severely wounded servicemembers. J Rehabil Res Dev. (2011) 48(2):89–102. 10.1682/JRRD.2010.04.0070 21480084

[41] Waid-Ebbs JK Wen PS Graham DP Ray K Leroux AJ O'Connor MK Factor structure of the community reintegration of service-members (CRIS) in veterans with blast-related mild traumatic brain injury. J Appl Meas. (2018) 19(4):363–9. PMID: 30433880

[42] Resnik L Borgia M Ni P Pirraglia PA Jette A. Reliability, validity and administrative burden of the community reintegration of injured service members computer adaptive test (CRIS-CAT)”. BMC Med Res Methodol. (2012) 12:145. 10.1186/1471-2288-12-145 22984850 PMC3528459

[43] Green A Felmingham K Baguley IJ Slewa-Younan S Simpson S. The clinical utility of the beck depression inventory after traumatic brain injury. Brain Injury. (2001) 15(12):1021–8. 10.1080/02699050110074187 11712948

[44] Beck AT Steer RA Brown G. Beck depression inventory–II. Psychol Assess. (1996):11.

[45] Weathers F Huska J Keane T. The PTSD checklist military version (PCL-M). Boston, MA: National Center for PTSD (1991). 42.

[46] Weathers FW Litz BT Herman DS Huska JA Keane TM. The PTSD checklist (PCL): reliability, validity, and diagnostic utility. Annual convention of the international society for traumatic stress studies. San Antonio, TX (1993).

[47] Davison AC Hinkley DV. Bootstrap methods and their application. Cambridge: Cambridge university press (1997).

[48] Cohen J. A power primer. Psychol Bull. (1992) 112(1):155. 10.1037/0033-2909.112.1.155 19565683

[49] R Core Team. R: a language and environment for statistical computing. Vienna, Austria: R Foundation for Statistical Computing (2020). URL https://www.R-project.org/

[50] Vanderploeg RD Curtiss G Belanger HG. Long-term neuropsychological outcomes following mild traumatic brain injury. J Int Neuropsychol So. (2005) 11(3):228–36. 10.1017/S1355617705050289 15892899

[51] Schneiderman AI Braver ER Kang HK. Understanding sequelae of injury mechanisms and mild traumatic brain injury incurred during the conflicts in Iraq and Afghanistan: persistent postconcussive symptoms and posttraumatic stress disorder. Am J Epidemiol. (2008) 167(12):1446–52. 10.1093/aje/kwn068 18424429

[52] Otis JD McGlinchey R Vasterling JJ Kerns RD. Complicating factors associated with mild traumatic brain injury: impact on pain and posttraumatic stress disorder treatment. J Clin Psychol Med Settings. (2011) 18(2):145–54. 10.1007/s10880-011-9239-2 21626354

[53] Troyanskaya M Pastorek NJ Scheibel RS Petersen NJ McCulloch K Wilde EA Combat exposure, PTSD symptoms, and cognition following blast-related traumatic brain injury in OEF/OIF/OND service members and veterans. Mil Med. (2015) 180(3):285–9. 10.7205/MILMED-D-14-00256 25735018

[54] Ryan-Gonzalez C Kimbrel NA Meyer EC Gordon EM DeBeer BB Gulliver SB Differences in post-traumatic stress disorder symptoms among post-9/11 veterans with blast- and non-blast mild traumatic brain injury. J Neurotrauma. (2019) 36(10):1584–90. 10.1089/neu.2017.5590 30511882

[55] Kaplan GB Leite-Morris KA Wang L Rumbika KK Heinrichs SC Zeng X Pathophysiological bases of comorbidity: traumatic brain injury and post-traumatic stress disorder. J Neurotrauma. (2018) 35(2):210–25. 10.1089/neu.2016.4953 29017388

[56] Daniels JK McFarlane AC Bluhm RL Moores KA Clark CR Shaw ME Switching between executive and default mode networks in PTSD: alterations in functional connectivity. J Psychiatry Neurosci. (2010) 35(4):258. 10.1503/jpn.090175 20569651 PMC2895156

[57] Lanius RA Bluhm RL Coupland NJ Hegadoren KM Rowe B Theberge J Default mode network connectivity as a predictor of post-traumatic stress disorder symptom severity in acutely traumatized subjects. Acta Psychiatr Scand. (2010) 121(1):33–40. 10.1111/j.1600-0447.2009.01391.x 19426163

[58] Shoulson I Wilhelm EE Koehler R. Cognitive rehabilitation therapy for traumatic brain injury: evaluating the evidence. Washington, DC: National Academies Press (2012).

[59] Hardy M Kennedy J Reid M Cooper D. Differences in PTSD, depression, and attribution of symptoms in service members with combat versus noncombat mild TBI. J Head Trauma. (2019) 35(1):37–45. 10.1097/HTR.0000000000000486 31033746

[60] Stamenova V Levine B. Effectiveness of goal management training(R) in improving executive functions: a meta-analysis. Neuropsychol Rehabil. (2019) 29(10):1569–99. 10.1080/09602011.2018.1438294 29540124

[61] Stiers W Carlozzi N Cernich A Velozo C Pape T Hart T Measurement of social participation outcomes in rehabilitation of veterans with traumatic brain injury. J Rehabil Res Dev. (2012) 49(1):139–54. 10.1682/JRRD.2010.07.0131 22492344

[62] Miotto EC Evans JJ de Lucia MC Scaff M. Rehabilitation of executive dysfunction: a controlled trial of an attention and problem solving treatment group. Neuropsychol Rehabil. (2009) 19(4):517–40. 10.1080/09602010802332108 18766984

[63] Valls-Serrano C Caracuel A Verdejo-Garcia A. Goal management training and mindfulness meditation improve executive functions and transfer to ecological tasks of daily life in polysubstance users enrolled in therapeutic community treatment. Drug Alcohol Depend. (2016) 165:9–14. 10.1016/j.drugalcdep.2016.04.040 27246405

[64] Spikman JM Boelen DH Lamberts KF Brouwer WH Fasotti L. Effects of a multifaceted treatment program for executive dysfunction after acquired brain injury on indications of executive functioning in daily life. J Int Neuropsychol Soc. (2010) 16(1):118–29. 10.1017/S1355617709991020 19900348

[65] Levine B Schweizer TA O'Connor C Turner G Gillingham S Stuss DT Rehabilitation of executive functioning in patients with frontal lobe brain damage with goal management training. Front Hum Neurosci. (2011) 5:9. 10.3389/fnhum.2011.00009 21369362 PMC3043269

[66] Tornas S Lovstad M Solbakk AK Evans J Endestad T Hol PK Rehabilitation of executive functions in patients with chronic acquired brain injury with goal management training, external cuing, and emotional regulation: a randomized controlled trial. J Int Neuropsychol Soc. (2016) 22(4):436–52. 10.1017/S1355617715001344 26812574

